# Aqueous Two Phase System Assisted Self-Assembled PLGA Microparticles

**DOI:** 10.1038/srep27736

**Published:** 2016-06-09

**Authors:** Nitish Yeredla, Taisuke Kojima, Yi Yang, Shuichi Takayama, Mathumai Kanapathipillai

**Affiliations:** 1 University of Michigan-Dearborn, Department of Mechanical Engineering, 4901 Evergreen Road, Dearborn, MI 48128, USA; 2 Macromolecular Science and Engineering Program, University of Michigan Ann Arbor, Ann Arbor, MI 48109, USA; 3 University of Freiburg, Department of Microsystems Engineering Chemistry and Physics of Interfaces, Georges-Koehler-Allee 103, Freiburg, Germany, 79110; 4 Biomedical Engineering Department, University of Michigan Ann Arbor, Ann Arbor, MI 48109, USA

## Abstract

Here, we produce poly(lactide-co-glycolide) (PLGA) based microparticles with varying morphologies, and temperature responsive properties utilizing a Pluronic F127/dextran aqueous two-phase system (ATPS) assisted self-assembly. The PLGA polymer, when emulsified in Pluronic F127/dextran ATPS, forms unique microparticle structures due to ATPS guided-self assembly. Depending on the PLGA concentration, the particles either formed a core-shell or a composite microparticle structure. The microparticles facilitate the simultaneous incorporation of both hydrophobic and hydrophilic molecules, due to their amphiphilic macromolecule composition. Further, due to the lower critical solution temperature (LCST) properties of Pluronic F127, the particles exhibit temperature responsiveness. The ATPS based microparticle formation demonstrated in this study, serves as a novel platform for PLGA/polymer based tunable micro/nano particle and polymersome development. The unique properties may be useful in applications such as theranostics, synthesis of complex structure particles, bioreaction/mineralization at the two-phase interface, and bioseparations.

Aqueous two-phase systems (ATPSs) have been studied over the past decades due to its potential in bioseparation, high throughout assays, microfluidics, diagnostics, and bioreactors^1^,^2^,^3^,^4^,^5^,^6^,^7^,^8^. One of the most widely studied ATPS is the polyethylene glycol (PEG)/dextran system^9^. Many other ATPSs have been reported including ATPSs that utilize block co-polymers^10^,^11^,^12^,^13^. Among them, thermo responsive polymers can be used in place of the commonly used PEG in dextran–PEG two-phase systems and, therefore, offer additional tunable properties^12^. More specifically, ethylene oxide (EO)-propylene oxide (PO) random copolymers are water soluble, and separate into polymer-rich liquid crystalline phases and a water phase depleted of polymer above a lower critical solution temperature (LCST)^14^. Among the thermoresponsive polymers, Pluronic F127 has been widely used in biomedical applications^15^,^16^. Further, Pluronic F127 has a LCST around physiological temperatures, and has been shown to form various self-assembling structures for use in template-directed synthesis of organic and inorganic structures^17^,^18^. Here, we take advantage of the tunable behavior of Pluronic F127 together with its ATPS system formation capability to produce PLGA particles with combinations of desirable properties that are difficult to integrate into a single particle otherwise.

Several methods have been used to develop PLGA nano and micro particles over the last several decades^19^,^20^,^21^,^22^,^23^. Among them, emulsion, precipitation, spray drying, and dialysis methods are the most frequently used methods for the formation of particles^19^,^24^,^25^,^26^. While these methods have been used routinely, several limitations exist with the conventional methods. The drawbacks include: extensive organic solvent usage, poor encapsulation of hydrophilic and small molecule drugs, inability to form PLGA based particles with unique morphological and tunable properties^24^,^25^. ATPSs, due to its well-defined phase separation and resulting compartmentalization, could be a useful tool for templating polymer self-assembly and subsequent particle formation at the ATPS interphase. Further the method may afford particles with novel properties that solve some of the problems in conventional particle formation procedures. Zhang *et al.*^27^ recently reported the PEG/dextran ATPS phase-guided assembly of PEG-Polycaprolactone (PCL) and DEX-PCL polymersomes. Ma *et al.*^28^ reported the fabrication of PEG-DA/dextran microgel particles with complex shape morphologies. Although the well-studied PEG/dextran ATPS has been utilized in many applications, ATPSs assisted polymeric particle production is still in the early stages. Further, to our knowledge, until now F127/dextran ATPSs have been used for temperature responsive bioseparations of proteins^12^, but its potential in other applications have not been explored.

Here in this study, we embark on a new method of forming PLGA based particles using the Pluronic F127/dextran aqueous two-phase system to address some of the above-mentioned limitations with the conventional methods in polymeric particle production. By utilizing Pluronic F127-containing ATPS, we are able to form, PLGA based microparticles with unique morphologies and temperature responsive properties. Pluronic F127/dextran solutions above certain concentrations, exhibit phase separation by partition of Pluronic F127 in a dextran rich outer matrix. When the PLGA polymer is emulsified in the ATPS, the polymer self-assembles and forms microparticles. We studied the microparticle properties as a function of polymer composition, temperature, and stirrer speed. Depending on the ATPS/PLGA composition, the particles either formed a core-shell morphology or a hybrid microparticle structure. The temperature and stirrer speed influenced the particle size and morphological properties. Further due to the LCST properties of the Pluronic F127, the particles exhibit temperature responsive properties. These studies demonstrate a method to regulate the properties of microparticles by the Pluronic F127/dextran ATPS template guided self-assembly. The particles also showed no significant cytotoxicity effects. This proof of concept method of producing PLGA based particles will open new design and concept of forming nano and micro polymeric and liposome particles with novel physicochemical, and stimuli responsive properties that could be applied in drug delivery and other relevant biomedical applications.

## Results and Discussion

**Figure 1:**
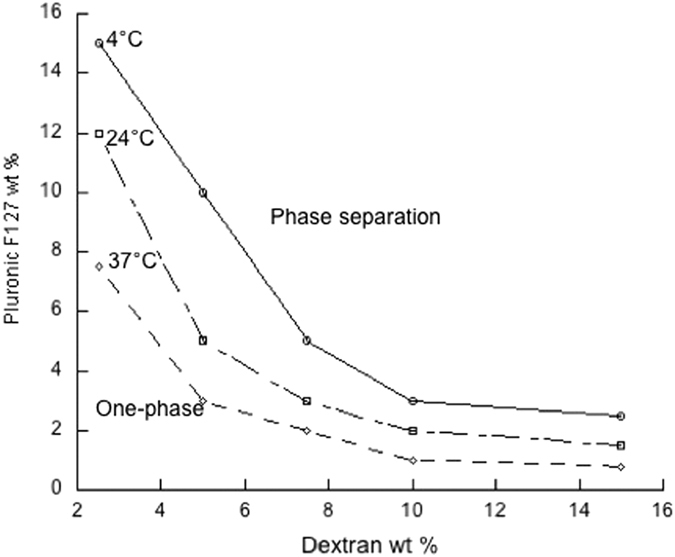
(**A**) Pluronic F127/dextran biphasic diagram at three different temperatures exhibiting thermoresponsive phase separation. (**B**) Phase separation of 3% Pluronic F127/10% dextran at 4 °C, 24 °C and 37 °C.

Pluronic F127, triblock copolymer composed of poly(ethylene oxide)-*b*-poly(propylene oxide)-*b*-poly(ethylene oxide) (PEO-PPO-PEO) has a weight percentage of 70% PEO and 30% PPO with an overall molecular weight of 12, 600. Below a certain temperature, which is known as the critical micelle temperature (CMT), both ethylene and propylene oxide blocks are hydrated and PPO is relatively soluble in water. With increase in temperature and concentration, PPO chains become less soluble, phase separates, resulting in liquid crystalline phases^14^. Hence, in addition to the ATPS phase induced separation, Pluronic F127/dextran also exhibit temperature responsive phase separation as a function of concentration. The Pluronic F127/dextran aqueous phase separation diagram is shown in Fig. 1. At physiological temperatures, due to the LCST properties of Pluronic F127, phase separation occurs at low concentrations. The phase separation of Pluronic F127/Dextran, together with the liquid crystalline phase behavior of Pluronic F127 presents an interesting system to produce particles with unique properties through ATPS template assisted self assembly.

**Figure 2:**
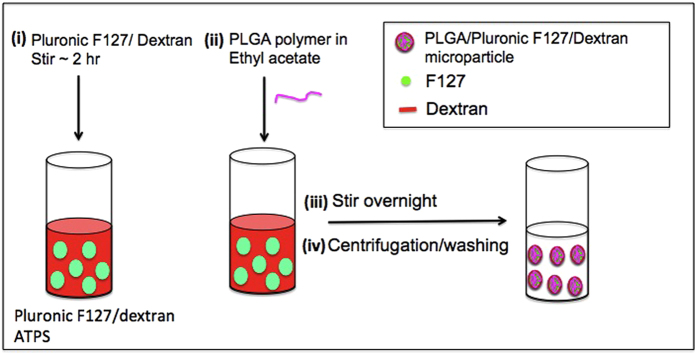
Schematic of the PLGA based microparticle production using Pluronic F127/dextran ATPS guided self-assembly. Production parameters temperature, PLGA concentration, dextran molecular weight, and the stirrer speed are varied to obtain particles with unique morphologies and characteristics.

The schematic in Fig. 2 shows how ATPS assisted self-assembly can be used to produce microparticles. For the study, dextran and Pluronic F127 concentrations were chosen to fall on or near the phase separation concentrations at room temperature. Three different final concentrations of PLGA (0.0625%, 0.125% and 0.25%) were used to study the effect of PLGA concentration on the morphology and size of the particles formed. PLGA polymer was dissolved in ethyl acetate, and the solution was dropped at a constant rate in to the ATPS and vortexed briefly to allow emulsion formation. At low concentrations of PLGA, due to the relatively higher solubility of ethyl acetate in water, and the amphiphilic properties of Pluronic F127, it is anticipated that the hydrophobic PLGA preferably partition into the Pluronic F127 phase, and separates from the hydrophilic dextran rich phase. At high concentrations of PLGA, no clear partitioning is expected due to the possibility of multiple emulsion formation. The emulsified ATPS/PLGA solution was then gently stirred for 12 hours, to allow adequate time for the formation of microparticles by the ATPS guided assembly. The particles were then purified by centrifugation. Microparticles with different morphology and properties are obtained depending on the production conditions.

**Figure 3:**
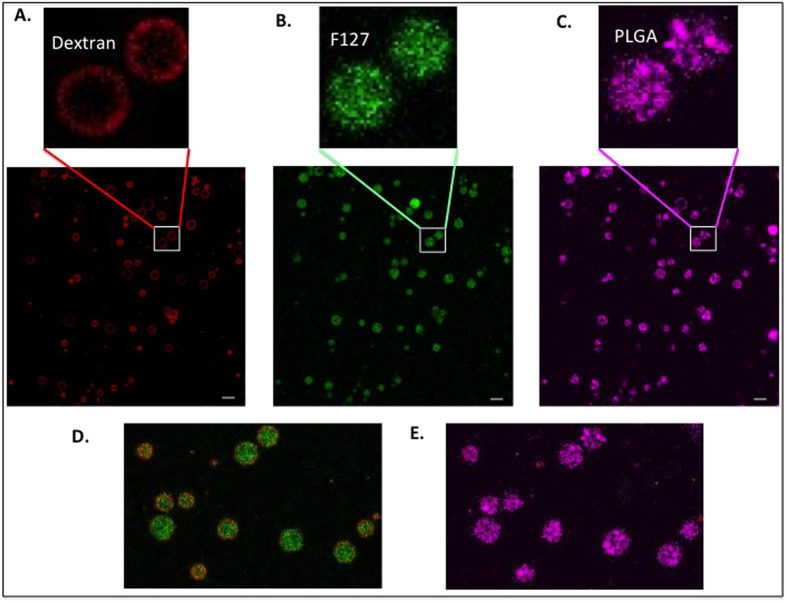
Three-color fluorescent micrographs of microparticle prepared from a mixture containing 0.0625% PLGA, 2% Pluronic F127/10% dextran. The fluorescence is from TRITC-dextran (red), FITC-Pluronic (green), and Cy5-PLGA (cyan). The dextran polymers form an outer shell whereas the Pluronic and PLGA polymers are concentrated in the core. Scale bar 10 μm.

**Figure 4:**
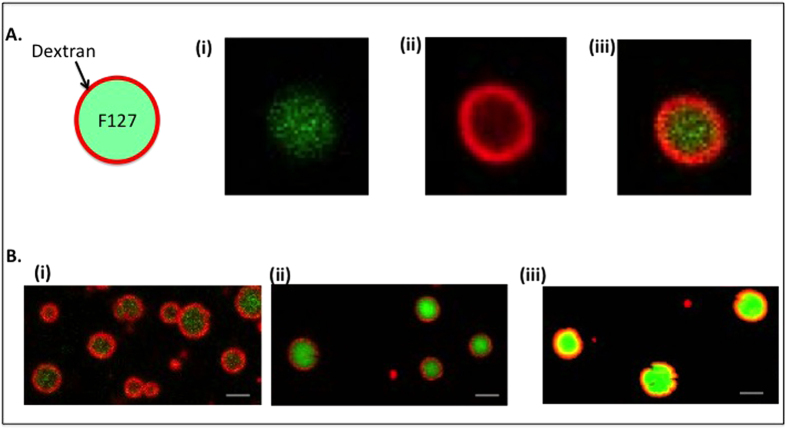
The formation of a core-shell structure is robust across a wide range of ATPS compositions. (**A**) Partlcle morphology at 2% Pluronic F127/10% dextran, and PLGA concentration of 0.0625%. (**B**) Here the PLGA concentration is held at 0.0625%, but the concentration of Pluronic F127 and dextran are varied as follows. (**B**) (i) 2% Pluronic F127/10% dextran (ii) 5% Pluronic F127/10% dextran (iii) 5% Pluronic F127/5% dextran ATPS compositions. Scale bar 10 μm.

Figure 3 shows confocal images of the microparticles produced with 0.0625% PLGA, and 2%Pluronic F127/10% dextran ATPS at room temperature. The particles exhibited a core shell morphology composed of a PLGA backbone matrix, dextran concentrated in the shell, and F127 concentrated in the core (Fig. 3D,E). The particle size ranged from 2–10 μm with an average size around 7 μm (Fig. S1). To examine how ATPS composition affects microparticle morphology, a single PLGA concentration was subjected to three different combinations of Pluronic F127/dextran ATPS compositions. All three exhibited similar core/shell morphology as shown in Fig. 4. Unlike PEG/dextran where an inverse phase separation is observed with increased PEG/dextran ratio^6^, Pluronic F127/dextran always phase separated with a dextran rich outer phase. This may be due to the amphiphilic nature of Pluronic F127, with the hydrophobic polypropylene oxide and hydrophilic polyethylene oxide structure, forming a Pluronic F127 dispersed phase within a continuous dextran phase. While the overall morphology is not influenced by the F127/dextran composition, the microparticle composition changes according to the F127/dextran ATPS composition as evidenced by NMR spectroscopy (Fig. S2). Comparing the peak areas in respect to the PLGA methyl group (-CH_3_) chemical shift at 1.66 pm, the peak ratio of the CH and CH_2_ functional groups (~4.5–5 ppm) of dextran/PLGA to that of the Pluronic F127 chemical shift around ~3.7 ppm increases for the F127/dextran ATPS with higher dextran content.

**Figure 5:**
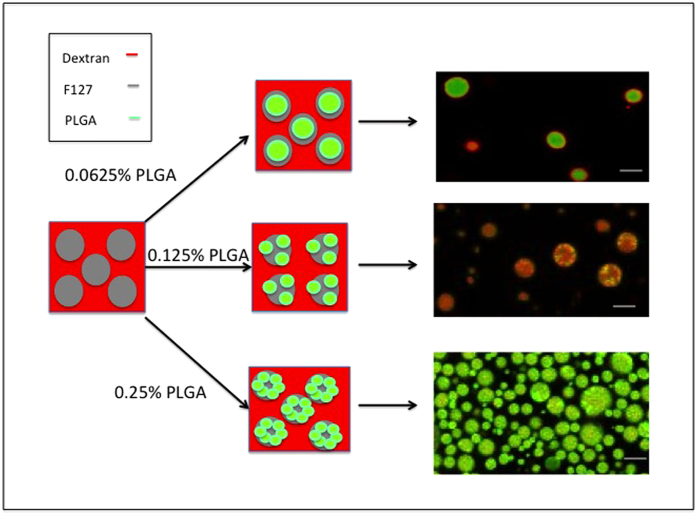
Influence of PLGA concentration on microparticle morphology with 5% Pluronic F127/10% dextran ATPS composition. The three different PLGA concentrations are (i) 0.0625% PLGA. (ii) 0.125% PLGA. (iii) 0.25% PLGA. The lower concentrations of PLGA yield core-shell particle morphology, while the higher concentrations exhibit hybrid/composite microparticles structures. Scale bar 10 μm.

**Figure 6:**
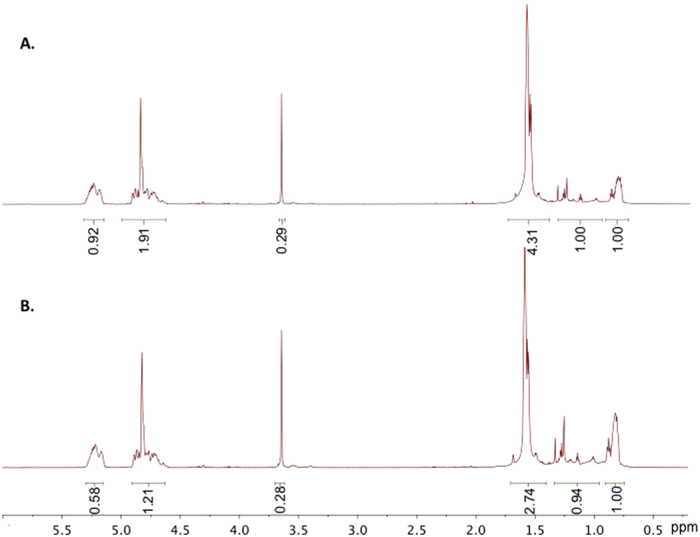
NMR spectra of microparticles comprising 5% Pluronic F127/10% dextran ATPS with PLGA concentrations of A) 0.125% PLGA. B) 0.0625% PLGA. The peak areas were normalized relative to the chemical shift (-OCH_2_CHCH_3_O) of Pluronic F127 around 1 ppm. The microparticle with higher composition of PLGA (**A**) exhibit increase in PLGA signal peaks corresponding to chemical shifts at 1. 66 ppm (-CH_3_), 5.08 ppm (-CH), and 4.99 pm (-CH_2_) compared to microparticle with lower PLGA content (**B**).

While ATPS composition had minimal effects on microparticle morphology, PLGA concentration had a large effect. Depending on the concentration of the PLGA polymer, the microparticles either exhibit a core-shell morphology or a polymer blended composite structure. Figure 5 shows confocal images of microparticles formed from various PLGA concentrations and the proposed mechanism of how the different morphology arise. At lower PLGA concentrations, the particles were composed of a PLGA base matrix, dextran concentrated in the shell, and Pluronic F127 concentrated in the core. With increasing concentrations of the polymer, the dextran phase, disperses more into the PLGA/Pluronic F127 matrix, forming composite microparticles structure composed of PLGA/Pluronic F127/dextran. This may be due to the fact that at higher concentrations of PLGA, the polymer phase emulsifies with multiple ATPS droplets leading to multiple emulsion. The particles were also characterized by ^1^HNMR spectroscopy. Figure 6 show that the particle composition changes as a function of PLGA concentration. The peaks areas were normalized to that of the Pluronic F127 peak (-OCH_2_CHCH_3_O) at around 1 ppm. As can be seen from Fig. 6A, the increase in PLGA peak areas at 1.66 ppm (-CH_3_), 5.08 ppm (-CH), and 4.99 pm (-CH_2_) compared to Fig. 6B confirm that the composition of the particle contain more PLGA with increasing PLGA concentration.

**Figure 7:**
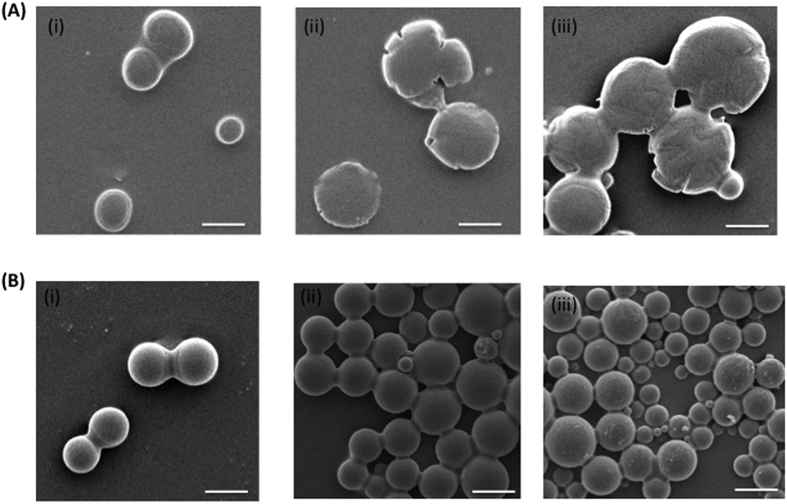
Influence of temperature on microparticle morphology comprising 5% Pluronic F127/10% dextran ATPS composition with 3 different PLGA concentrations. (i) 0.0625% PLGA. (ii) 0.125% PLGA. (iii) 0.25% PLGA. The two different temperature conditions used for the production are (**A)** Room temperature (**B)** 37 °C. The higher temperature conditions exhibit more defined spherical morphology and smaller size particles compared to the room temperature production conditions. Scale bar 10 μm.

Further, we studied the effect of processing temperature, stirrer speed, and molecular weights of the polymers, to see whether they could render additional novel particle properties. The particles exhibited controlled spherical morphology, with smaller sizes at high temperature processing as shown in the scanning electron microscopy (SEM) images (Fig. 7). This may be due to the lower interfacial tension, and the LCST behavior of Pluronic F127 at high temperatures that facilitate smaller emulsion droplets, and controlled self-assembly of the particles. This is also in agreement with Choi *et al*.’s findings on decrease in particle size of PLGA/Pluronic surfactant microparticles at higher temperatures^29^, and Alexandridis *et al*.’s study on the stabilization effect of Pluronic triblock copolymer structures at higher temperatures^30^. The effect of dextran molecular weight does not seem to have a significant effect on the morphology and size (Fig. S3 (ii)). On the other hand, particle formation with increased stirrer speed produced smaller size particles with distorted morphology (Fig. S3 (iii)), compared to the lower stirrer speed (300 rpm) that was used for the production of most of the particles in this study. The higher energy transferred at high stirring speed may have aided in smaller emulsion droplets and hence the smaller particle size. These results indicate high temperature and stirrer speed have significant influence on the particle morphology and size.

**Figure 8:**
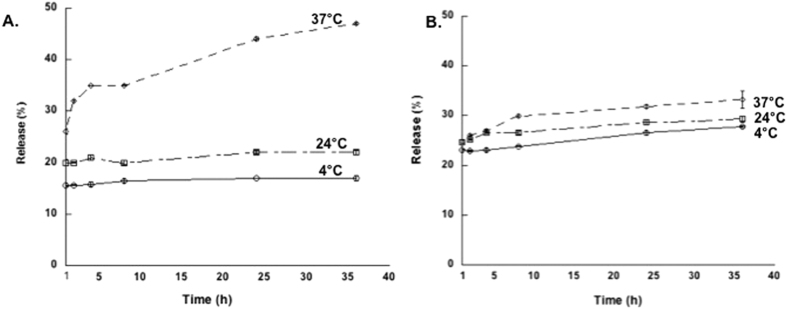
Drug release profile of microparticle composed of, 5% Pluronic F127/10% dextran, and 0.125% PLGA. (**A**) Hydrophilic rhodamine B release, and (**B**) hydrophobic coumarin-6 dye release, exhibiting temperature responsive release at 37 °C compared to room temperature, and 4 °C due to the LCST properties of Pluronic F127.

The PLGA microparticle structure composed of dextran and Pluronic F127, makes it possible for simultaneous incorporation of both hydrophilic and hydrophobic molecules. Furthermore, this endows the particles with thermoresponsive properties due to the LCST properties of Pluronic F127. To test its potential as a temperature stimuli responsive drug depot, particles were loaded with fluorescence dyes, and the loading and release studied. The release kinetics of a model hydrophilic dye (rhodamine B) and hydrophobic dye (coumarin-6) are shown in Fig. 8. The results show increase in release at 37 °C compared to room, and 4 °C indicating temperature responsiveness. This could be due to the fact that, the LCST behavior of Pluronic F127 around 37 °C^15^,^16^, facilitates phase separation, driving the Pluronic F127 to shrink and expel water and other soluble molecules from the microparticle. To confirm, that this is indeed due to Pluronic F127 LCST behavior and not due to the increase in diffusion at higher temperatures, we performed release studies with microparticles composed of lower Pluronic F127 content in the ATPS with the same amount of drug loading. The results show decrease in temperature responsive release (Fig. S4), compared to microparticle produced from higher Pluronic F127 content in the ATPS (Fig. 8), suggesting that Pluronic F127 influences the temperature responsive release. The studies also show that the hydrophilic drug release exhibited significant temperature responsive release properties compared to the hydrophobic drug. We have also studied the loading efficacy of the particles. The loading efficacy of hydrophilic, hydrophobic, and a large biomolecule are shown in Table 1. The hydrophobic coumarin-6 dye has a high loading efficacy with around 55% for the higher PLGA composition. However, the hydrophilic drug, showed lower loading, perhaps due to its affinity to the dextran phase, which is mainly concentrated in the core of the microparticle that is readily subject to hydration. To test, whether the particles affect cell viability, we performed XTT assays. SK-BR-3 breast cancer metastatic cells incubated with the microparticles did not show significant cytotoxicity as shown in Fig. S5. The results show promise of these microparticles as drug delivery vesicles and in other biomedical applications.

## Conclusion

This paper provides proof-of-concept of a two-phase system based, programmable, PLGA particle formation method. We illustrate promise of this method through preparation of biocompatible and biodegradable microparticles that are capable of incorporating hydrophobic, large biomolecules, and hydrophilic drugs under mild conditions, a feat that is difficult to achieve using conventional PLGA microparticle formation protocols. Additionally, the particles prepared demonstrate temperature-responsive drug release, presumably due to use of a temperature responsive polymer as one of the aqueous phase forming polymers. This novel method complements other existing protocols by enabling preparation of particles with distinct morphologies, drug loading capabilities, and tunable stimuli responsive properties that are difficult to achieve otherwise.

## Methods

### Materials

PLGA polymer (Mw 7–17 kDa, 50:50, acid terminated) was purchased from Evonik. Cy5-labelled PLGA was purchased from PolySciTech. Amine-PEG-FITC was purchased from Nanocs Inc. Dextran (500, and 10 kDa) was purchased from Pharmacosmos (Denmark), and (PEO-PPO-PEO)-NHS was purchased from Polymer Source Inc. All other materials were purchased from Sigma-Aldrich (St. Louis, MO).

### Phase diagram

First, appropriate quantities of Pluronic F127 and dextran were weighed and various concentrations of Pluronic F127 and dextran in water were prepared. The solutions were then mixed and equilibrated at room, 4 °C, and 37 °C temperatures to allow phase separation. The phase separation was monitored by simple visualization, and tube inversion technique and phase diagrams were generated for each temperature.

### Microparticle formulation

Pluronic F127/dextran ATPS was formed with 3 different combinations of Pluronic F127/dextran. ATPS solutions of 2% F127/10% dextran, 5% F127/10% dextran or 5% F127/5% dextran were used for the study. The ATPS solutions were stirred for 2 hours. PLGA was dissolved in small amount of ethyl acetate. Three different PLGA concentrations (0.0625%, 0.125% and 0.25%) were used for the study. The PLGA solutions were dropped slowly into dextran/Pluronic F127 ATPS solution, and stirred overnight. The solutions were centrifuged washed thrice. The purified particles were characterized with confocal microscopy and scanning electron microscopy (SEM).

### Confocal microscopy

Nikon A-1 spectral confocal at the UM-Ann Arbor microscopy image analysis laboratory (MIL) was used to image the morphology of the microparticles. For the dextran, about 0.2% of TRITC-500 dextran was used for labeling. For the Pluronic-F127, either 0.1% of PEO-PPO-PEO-NHS conjugated with NH2-PEG-FITC or trace amount of PEG-FITC was used. Either Cy5 labeled PLGA of a hydrophobic coumarin-6 dye was used to label the PLGA polymer. All samples were observed in microscope glass slides, capped with coverslips. Images were analyzed with ImageJ software.

### Scanning Electron Microscopy (SEM)

Scanning Electron microscopy images were obtained using AMRAY FEG-SEM at the university of Michigan Medical school Microscopy and Imaging Lab (MIL) facility. Samples were placed on carbon tapes adhesive substrates. The samples were then sputter coated with gold, and imaged at 5 kV using backscatter detector at 10 mm working distance.

### Nuclear Magnetic Resonance (NMR)

Proton NMR measurements were performed at University of Michigan Chemistry department NMR facility. Microparticles were dissolved in deuterated chloroform at 2 mg/ml, and NMR measurements were performed using Varian vnmr 700 instrument. The spectra were analyzed using MestReNova software.

### Drug release

The hydrophobic and hydrophilic small molecule dyes coumarin-6, and rhodamine B, were used as model drugs for the release studies. The hydrophobic coumarin-6 dye was dissolved with PLGA polymer and incorporated into the microparticles during emulsification process. Rhodamine-B dye was co-dissolved with dextran polymer, and Fitc-Albumin was co-dissolved with Pluronic-F127 during the microparticle production. The drug release was studied over a period of 72 hours. Particles were loaded with the dyes and the release were recorded at 1, 2, 4, 8, 24 and 36 hours at 4 °C, 24 °C and 37 °C. The drug release was quantified using a spectramax spectrophotometer in the lab.

### XTT assay

XTT assay purchased from ATCC was used to assess the cytotoxicity effects of the microparticles. For the study, metastatic breast cancer cell line SK-BR-3 from ATCC was used. 10^4^ cells were seeded in 96 well plates, and cells were incubated with particles with concentrations of 1 mg/ml, 0.1 mg/ml, 0.01 mg/ml. After 48 hours of incubation, XTT reagent was added to the cells, and the absorbance measurements were performed according to the XTT protocol provided by the manufactures.

### Statistical Analysis

Data were collected from three or more replicates for each experiment, and they are presented as mean ± standard error of the mean (SEM).

## Additional Information

**How to cite this article**: Yeredla, N. *et al.* Aqueous Two Phase System Assisted Self-Assembled PLGA Microparticles. *Sci. Rep.* **6**, 27736; doi: 10.1038/srep27736 (2016).

## Supplementary Material

Supplementary Information

## Figures and Tables

**Table 1:** Microparticle (MP) composition and loading efficacy of model drugs hydrophilic Rhodamine B, hydrophobic Coumarin-6, and biomolecule Fitc-Albumin as tabulated below.

Model Drug	MP composition on loading		
	F127/Dextran/PLGA (5:10:0625)	F127/Dextran/PLGA (5:10:0.125)	F127/Dextran/PLGA (5:10:0.25)
Rhodamine B	7.9 ± 0.3	8 ± 0.5	16.1 ± 0.6
Coumarin-6	36.1 ± 0.6	40 ± 2	55.8 ± 0.8
Fitc-Albumin	0.4 ± 0.01	1.54 ± 0.3	0.5 ± 0.2
