# Supplementary material for: Aqueous Two Phase System Assisted Self-Assembled PLGA Microparticles
Source: Sci Rep. 2016 Jun 9;6:27736. doi: 10.1038/srep27736 (PMC4899744; doi:10.1038/srep27736)
Supplement: Supplementary Information [file srep27736-s1.pdf]

Supporting Information

for *Scientific Reports*

Aqueous Two Phase System Assisted Self-Assembled PLGA Microparticles

Nitish Yeredla, Taisuke Kojima, Yi Yang, Shuichi Takayama, and Mathumai Kanapathipillai*

Figure S1

Particle size distribution of microparticle comprised of 0.0625% PLGA, 2% Pluronic F127/10% dextran.

Figure S2

NMR spectra of microparticles comprising 0.125% PLGA with ATPS compositions A) 5% F127/10% Dextran, and B) 5% F127/ 5% Dextran. The peak areas were normalized to the chemical shift (-CH_3) of PLGA around 1.6 ppm. The peak ratio of the CH and CH_2 functional groups (~ 4.5-5 ppm) of dextran/PLGA to that of the Pluronic F127 chemical shift around ~ 3.7 ppm, increases for the F127/dextran ATPS with higher dextran content(A).

Figure S3

Influence of dextran molecular weight, and stirrer speed on the morphology of the microparticle composed of 5% Pluronic F127/10% dextran, and 0.125% PLGA. i) 500 K dextran, stirrer speed (300 rpm) ii) 10 K dextran, stirrer speed (300 rpm) iii) 500 K dextran, stirrer speed (10,000 rpm). Scale bar 10 μm.

Figure S4

Rhodamine B drug release profile of microparticle composed of 2% Pluronic F127/10% dextran and 0.125% PLGA.

Figure S5

Effect of microparticle concentration on the cellular viability of SK-BR-3 metastatic breast cancer cells *in vitro*. Figure shows no significant toxicity of the microparticle (5% Pluronic F127/10% dextran, and 0.125% PLGA) at 0.001, 0.01 and 0.1 mg/ml concentrations.

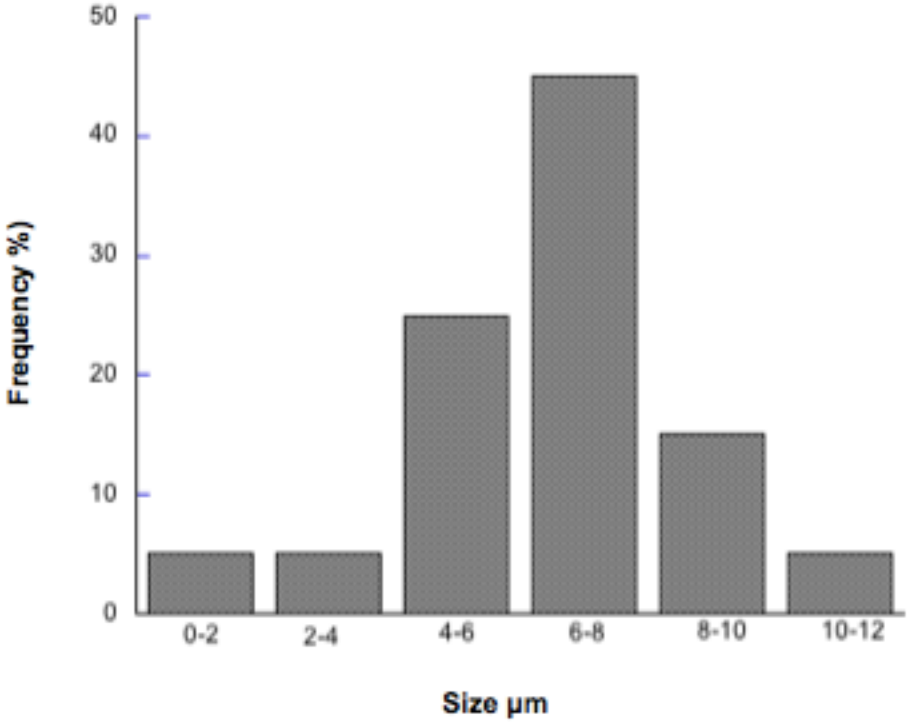

Figure S1

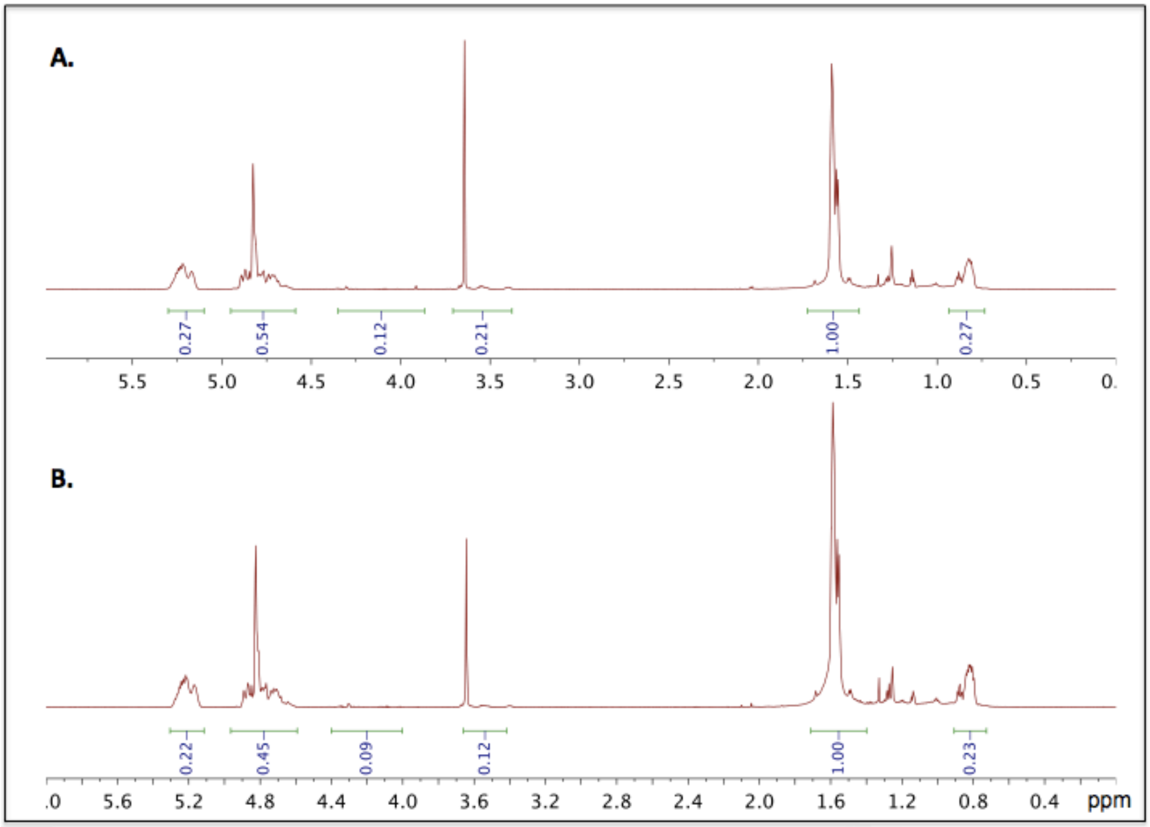
A.
0.27
0.54
0.12
0.21
1.00
0.27
5.5 5.0 4.5 4.0 3.5 3.0 2.5 2.0 1.5 1.0 0.5
B.
0.22
0.45
0.09
0.12
1.00
0.23
5.6 5.2 4.8 4.4 4.0 3.6 3.2 2.8 2.4 2.0 1.6 1.2 0.8 0.4 ppm

Figure S2

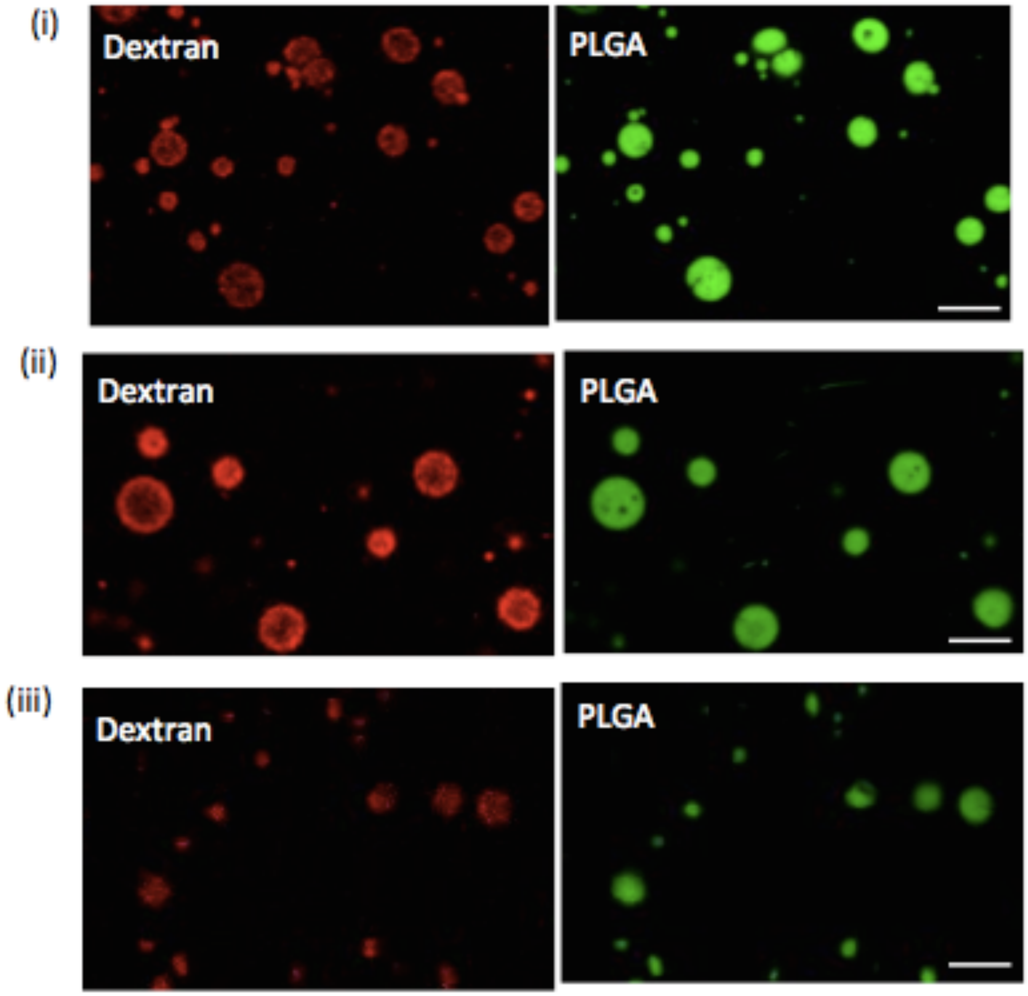

Figure S3

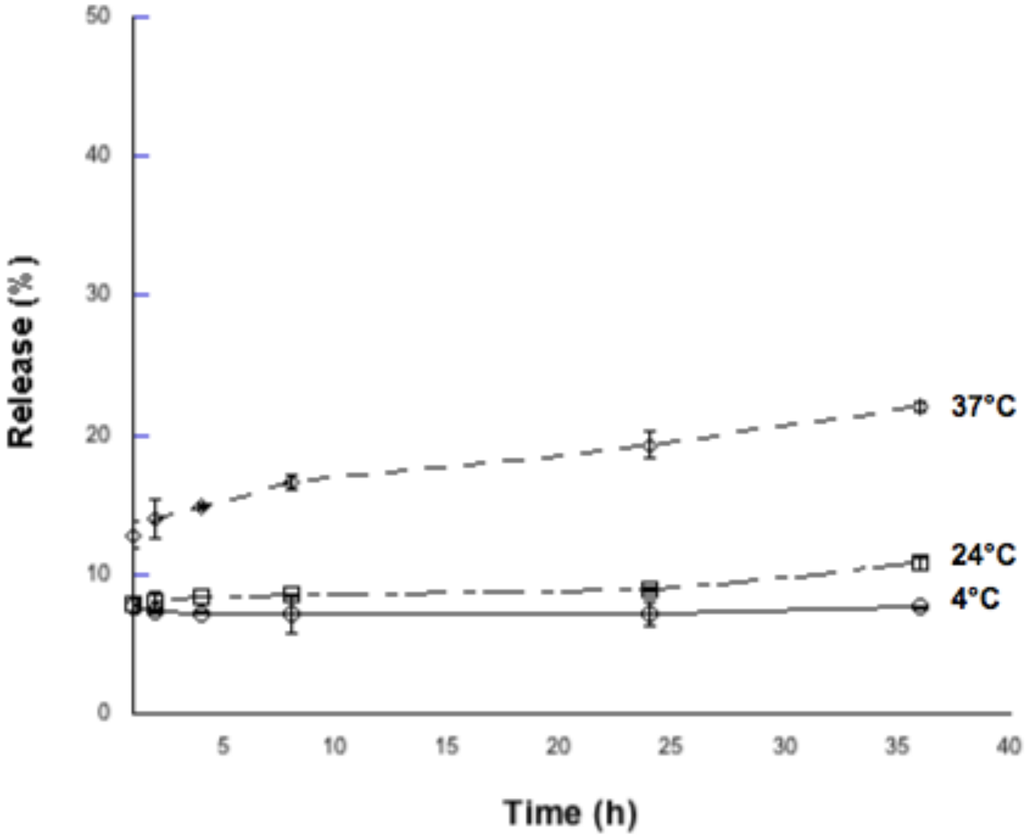

Figure S4

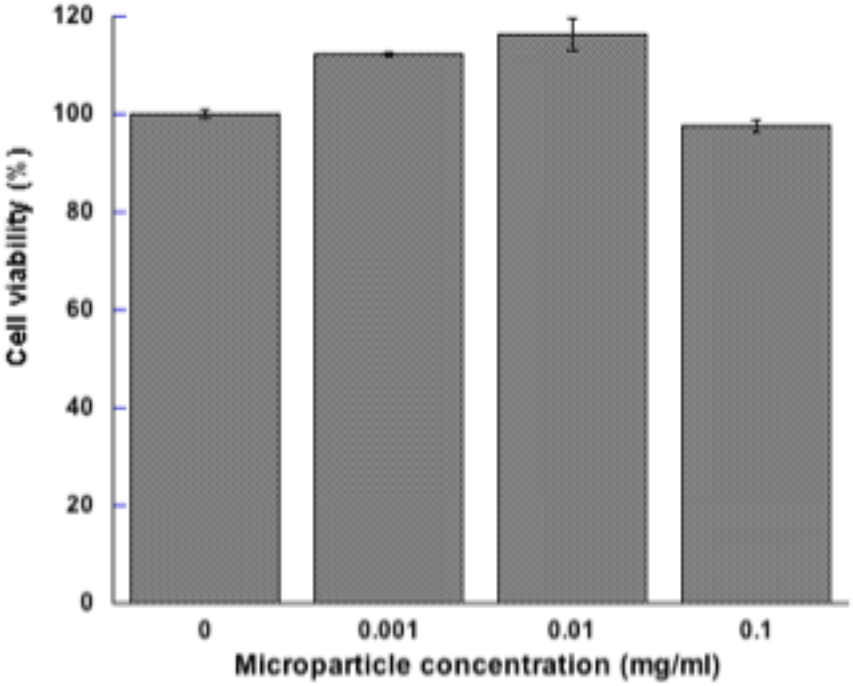

Cell viability (%)
120
100
80
60
40
20
0
0
0.001
0.01
0.1
Microparticle concentration (mg/ml)

Figure S5
